# Dataset for the long-term trade (1900–2021) and GDP (1960–2021) statistics of small island economies

**DOI:** 10.1016/j.dib.2024.110674

**Published:** 2024-06-26

**Authors:** Nicolas Lucic

**Affiliations:** a UMI SOURCE and CEMOI, Université de Versailles Saint Quentin en Yvelines – Paris Saclay, Bâtiment Vauban, Porte 607, 47 Boulevard Vauban, 78047 Guyancourt CEDEX, France; b UMI SOURCE and CEMOI, Université de la Réunion, CS, 92003, 15 Avenue René Cassin, Saint Denis CEDEX 9, 97400 Réunion, France

**Keywords:** Imports, Exports, Small Islands Developing States (SIDS), Economic development, Long term development, Independence, Affiliation, Colonial archives

## Abstract

This historical dataset regarding island and coastal economies has been built with the intent of making up for the gaps and shortcoming of the economic literature on long-term island development. This dataset gathers Imports, Exports, GDP, Population and Exchange Rate data on a yearly basis. The idea was to gather as much data as possible, with the reference period being 1900–2021 for foreign trade and 1960–2021 for GDP data. This dataset is relevant as it opens up new possibilities of long-term systematic comparisons between a great number of territories. Data were collected from international databases (UNCTAD, UN Statistics Division, UN Statistical Yearbook) when available. Some scholarly works were also used, most notably Mitchell [4] and Bulmer-Thomas [2]. When these sources were exhausted, the author went himself to the French Archives (INSEE) and the British National Archives (at Kew) to consult physical copies of colonial reports in order to find data prior to the 1950ʼs. Other islands’ data were reconstructed by contacting the local archives or statistical offices. For the foreign trade dataset, data are expressed in local currencies, then transformed to current USD, to constant 2005 USD equivalent and finally to constant 2005 USD per capita equivalent. These different levels of disaggregation are voluntarily left for each reader to use. For the GDP dataset, data are presented in current USD, current USD per capita and constant 2015 USD per capita equivalent. Different datasets have different levels of disaggregation. Some territories could not be included for practical reasons. The Italian islands are not included as the author could not go to the National Institute of Statistics (ISTAT) to consult the data in person. German island colonies in the Pacific could not be studied as the author does not understand the older German. Also, the India Office of the British Colonial Archives has been transferred to Qatar National Library, which scans and makes documents progressively available. Finally, the Western Pacific Archives were transferred to the University of Auckland Special Collections since 2002 and the author could not consult them. A coordinated effort by the research community could resolve these issues.

Specifications Table

**Table:** 

Subject	Economics, Econometrics and Finance Economic Development and Growth
Specific subject area	Study of long-term economic development in island territories where few macroeconomic data are usually available. This data paper fills in the aforementioned absence.
Type of data	Raw data, Analyzed data, Processed data.
Data collection	Data were collected from international databases online as well as physical archival material from various archives. Data were then transcribed into a dedicated excel sheet for each island. All available data for an island were reported on the aforementioned sheet. There was minimal filtration (a few incoherent data were excluded, such as reporting errors). At a later stage, data were transformed and normalized in current USD equivalent, then constant 2005 USD equivalent and finally per capita constant 2005 USD equivalent for international comparison purposes. The transformation methodology is detailed below and in comments found within the Excel sheets, in column titles.
Data source location	Archival data location below: · INSEE - Bibliothèque de l'INSEE Alain Desrosières · 88 Avenue Verdier, Montrouge, 92120 · France · Records of the Colonial Office, Commonwealth and Foreign and Commonwealth Offices; The National Archives, Kew · Bessant Dr., Richmond TW9 4DU · United Kingdom · British Library · 96 Euston Road, London, NW1 2DB · United Kingdom · Instituto Nacional de Estatistica · Avenida Antonio José de Almeida s/n, 1000-043 Lisboa · Portugal · Arquivo Historico Ultramarino · Calcada da Bao Hora 30, 1300-095 Lisboa · Portugal · National Institute of Statistics · Via Cesare Balbo 16, 00184 Rome · Italy
	· Statistics Denmark · Sankt Kjelds Plads 11, DK-2100 Copenhagen · Denmark · The Ohio State University Library · 1858 Neil Avenue Mall, Colombus, OHIO 43210 · United States · Ministry of Education - Department of Archives · Government Administration Building, Basement 30 Parliament Street, Hamilton HM 12 · Bermuda · National Statistics Office - Malta Blue Books · Lascaris, Valletta, VLT 2000 · Malta · Direcao Regional de Estatistica da Madeira · Calcada de Santa Clara 38, Funchal 9004-545 · Madeira, Portugal · Servico Regional de Estatistica dos Acores · Sede, Rua de Rocha 26/Rua do Salinas 1, Angra do Heroismo 9700-169 · Acores, Portugal · Newfoundland & Labrador Statistics Agency - Finance, Historical Statistics of Newfoundland and Labrador · 5 Mews Place, Saint John's, NL, A1B 4J6 · Newfoundland & Labrador, Canada · Collection IOR/L/PS/12/3890, Persian Gulf Administration Reports - Qatar National Library · District of Freedom, Education City, Al Luqta St, Doha · Qatar · Western Pacific Archive - Library of the University of Auckland · Princes Street, Auckland 1010 · New Zealand · Deutsches Kolonialblatt 1890-1921, Deutsches Zentrum Kulturgutverluste, https://www.proveana.de/de/literatur/deutsches-kolonialblatt-1890-1921 · Humboldtstrasse 12, 39112 Magdeburg · Germany
Data accessibility	Repository name: Recherche.data.gouv.fr > Université de Paris Saclay Data identification number: 10.57745/UA2LRO Direct URL to data: https://doi.org/10.57745/UA2LRO

## 1 Value of the Data

• This data paper aims at the first systematic exploration of long-term insular economics. Prior to this data paper, G. Bertram [1] has attempted to construct a smaller scale dataset for insular trade. It is the first and only dataset that attempts to cover island trade for at least 1900–2021 (and more if available) and GDP statistics for 1960–2021 when available.
• The dataset was deliberately constructed using various sources and their description. It has different levels of disaggregation, from data expressed in current local currency unit to their transformed equivalent in per capita 2005 constant USD values for trade (imports and exports) and 2015 constant USD for GDP. Researchers (mostly economists and historians) can use these data in their rawest form and transform them on their own initiative or they can choose to use the dataset with the transformations operated by the author (transformed to current USD, to 2005 constant USD or to per capita 2005 constant USD). The historical range varies from one territory to the other.
• This type of data has been used by the research community in development economics in order to make long term international comparisons. For example, Hoarau and Lucic [3] are partly based on this dataset. The most important novelty of this dataset is the systematic inclusion of islands that are affiliated to a metropolitan power (Réunion Island with France for instance). Their inclusion has been a challenge for prior researchers as their trade and GDP data are not widely available in international databases (World Development Indicators, UN Data, …) and only a few, mostly regionally based studies (Bulmer-Thomas, Bertram) have reported on affiliated islands.
• This dataset is also the first and only one that systematically gathers population data for comparison purposes. It is particularly important as population data for affiliated islands are especially hard to come by on the internet. Usually, population data is derived from various historical censuses which are held by the national archival funds that have to be visited in person. Centralization of this type of data is thus crucial for international comparison purposes.
• Finally, this dataset systematically gathers GDP data, for both independent (widely available) and affiliated (very poor availability) territories. It is important to note that the range is 1960–2021 and varies from one territory to the other. The GDP dataset is the first one with a systematic and historical account of island territories around the world. It covers all available data, up to date.

## 2 Background

The datasets, both the trade and the GDP one, come from practical hurdles within the research field of development economics. Indeed, the literature focusing on island studies and island development has found that affiliated territories have better results than independent ones. A part of the literature has attempted to explain this divergence by pointing to the costs of independence. To do so, data going back to 1960′s-70′s have been used in various studies. Bertram [1] uses another indicator, namely imports per capita in 2005 constant USD to go back to 1900 and the period prior to the independence process, starting in the 1950's for island territories. To date, it is the only macroeconomic indicator that can be used to approximate the living standards within island economies, before the 1960′s (see comments in the repository) [5] and in a continuous manner, with the aim of having long term studies.

Thus, this dataset aims at systematizing and grouping all trade and GDP data available for island economies with the goal of deepening the understanding of why affiliated islands perform better than independent ones. Lastly, one of the purposes of this dataset is to analyze divergences during the colonial period.

## 3 Data Description

There are three separate data files within the repository [5]. The first one, named ‘*NLucic_SIHTD_Details_of_Foreign_Trade_per_Island_with_sources-14032024*′, holds 80 sheets in Excel format. Every single sheet details all available sources for trade data (imports and exports). There can be different sources for the reconstruction of raw data. There are 80 sheets total, one per territory.

Every sheet follows the same methodology: the first columns gather all available trade data in local currency unit. There can be several different local currency units with associated data. For example, islands under French colonial administration can have data expressed in (ancient) French Francs, in CFA Francs, in CFP francs, in New French Francs and in Euros. Every source is indicated in detailed comments that can be found in column titles. Comments either contain sources and the years covered by each source or methodology when needed. Comments can cover both sources and methodology if needed. The reference period covered is 1900–2021 but when the author was able to find data that went back further than 1900, it is also included in the sheets.

After all raw data in local currency units are reported, the same methodology is followed. The raw data in local currency are converted to current USD by using the official exchange rate (sources are indicated in comments) LCU to USD. Once data are converted to current USD, two columns are systematically present: ‘Total population’ or an appropriate column title that displays different population data (with detailed sources in comments) and ‘Value of the US inflation understood as the 1 USD expressed in 2005 USD for the corresponding year’ which is the value by which one needs to multiply the current trade values to suppress the inflation phenomenon and transform series in constant 2005 USD equivalent values. This methodology can be criticized as the multiplication factor represents the USD inflation and is applied to every territory regardless of historical local inflation dynamics but the author has checked for alternative methods. Indeed, the author has tried to estimate the local inflation rates and then convert the values from 2005 LCU to 2005 USD. The results are very close, meaning that the economic hypothesis of exchange rates between two currencies being the expression of differentiated inflation rates seems valid. Regardless, different researcher can construct their own inflation-negating indexes and simply take the trade values in current USD equivalent.

The author, when applicable, puts the most transformed data furthest to the right of each sheet. Usually, the final four columns are named, respectively, ‘*Imports in constant 2005 USD equivalent*’, ‘*Exports in constant 2005 USD equivalent*’, ‘*Imports per capita in 2005 USD equivalent*’ and ‘*Exports per capita in constant 2005 USD equivalent*’, or a very close variation.

The next data file is named ‘*NLucic_SIHTD_Continuous_Foreign_Trade_series_1900-2021-14032024*′ and is destined to be used by researchers only looking for complete trade series for 1900–2021. Only islands that have complete series of both imports and exports from 1900 to 2021 (with a few exceptions such as data in 2020 or 2021 that could not be updated) were included in this file. It should be thought of as an excerpt of the much larger and detailed first file, cited above.

Indeed, this second file has six sheets in Excel format, in order:

- *Imports in current USD*: 49 columns, column A lists years in a continuous manner; columns B to AW contain individual territories, more precisely, it contains territories as an institution (a good example are the Netherlands Antilles, which included six different islands under the same institutional arrangement). Each datum represents imports (or exports) of corresponding islands for a given year, already converted to USD. Detailed sources and justifications for the construction of this dataset are duly given in the first dataset named ‘*NLucic_SIHTD_Details_of_Foreign_Trade_per_Island_with_sources-14032024*′.
- *Exports in current USD*: 49 columns, column A lists years in a continuous manner; columns B to AW contain individual territories, more precisely, it contains territories as an institution (a good example are the Netherlands Antilles, which included six different islands under the same institutional arrangement). Each datum represents imports (or exports) of corresponding islands for a given year, already converted to USD. Detailed sources and justifications for the construction of this dataset are duly given in the first dataset named ‘*NLucic_SIHTD_Details_of_Foreign_Trade_per_Island_with_sources-14032024*′.
- *Imports in constant 2005 USD*: 49 columns where column A lists years in a continuous manner; columns B to AW contain yearly data for individual territories, listed in the same order as sheets prior. All data are taken from the first Excel document named above.
- *Exports in constant 2005 USD*: 49 columns where column A lists years in a continuous manner; columns B to AW contain yearly data for individual territories, listed in the same order as sheets prior. All data are taken from the first Excel document named above.
- *Imports pC in constant 2005 USD*: 49 columns where column A lists years in a continuous manner; columns B to AW contain yearly data for individual territories, listed in the same order as sheets prior. All data are taken from the first Excel document named above.
- *Exports pC in constant 2005 USD*: 49 columns where column A lists years in a continuous manner; columns B to AW contain yearly data for individual territories, listed in the same order as sheets prior. All data are taken from the first Excel document named above.

The third and last file is named ‘*NLucic_SIHTD_GDP_1960–2021_with_sources-14032024*′ holds three sheets. Every sheet details all available sources for GDP data. The reference period for this file is 1960–2021. There can be different sources for the reconstruction of raw data.

The sheets, in this file, are broken down as follows:

- *Current GDP USD*: 87 columns, where A lists years in a continuous manner; columns B to CI represent each individual territory. Data are all presented in USD equivalent. Every source is indicated in detailed comments that can be found in column titles. The level of disaggregation in this sheet is lower than what was presented in the first document. There is a degree of transformation in this sheet where original raw GDP data was transformed to current USD using the average yearly exchange rate from Local Currency Unit (LCU) to USD. For this dataset, a lot of territories share the same original data which taken from the World Development Indicators database, compiled by the World Bank.^1^ The WDI covers independent territories and the most autonomous island territories. Also, data for various islands can be found in UN Data,^2^ from the UN Statistics Division, National Accounts Estimates of Main Aggregates, series Per capita GDP at current prices – USD. Finally, one other common source of data for island territories is Victor Bulmer-Thomas’ work^3^ on Caribbean countries. All of the details regarding which year is covered by which source can be found in the comments within the column titles.
- *Current GDP pC USD*: 87 columns, where A lists years in a continuous manner; columns B to CI represent each individual territory. Data are all presented in per capita USD equivalent. Every source is indicated in detailed comments that can be found in column titles. For the independent and the most autonomous island territories, the WDI and UN Data cited above produce GDP per capita data. When available, those were selected. If unavailable, data compiled in the previous sheet has been divided by total population for each individual year. The details of total population reconstruction has been indicated in the comments within the column titles, alongside other detailed sources.
- *Constant 2015 GDP pC USD*: 87 columns, where column A lists years in a continuous manner; column B lists the compiled USD deflator (base 1 in 2015) taken from the World Development Indicators, cited above; and column C to CJ represent each individual territory. Data are all presented in constant 2015 GDP per capita USD equivalent. There is no need for detailed comments in column titles on this sheet as every source has already been cited in previous sheets. Data displayed in this sheet is the corresponding data in the sheet ‘*current GDP pC USD*’ and multiplied by the compiled deflator value for the year.

## 4 Experimental Design, Materials and Methods

Besides Bertram [1], there was no other example of researchers looking for historical trade data for island territories in particular. There has been other research conducted on historical statistics and they have sometimes included some island territories, for example Bulmer-Thomas [2] or Mitchell [4]. The former systematically reports on Caribbean economies since 1900 whereas the latter has a global scope, but only reports on the most important colonies (those for which data were relatively easy to access). Important to note that Bertram did collect some data on a few pacific islands but never published them. The author has received them directly from Bertram. They can be shared by the author upon demand. Besides this data paper, to the best of the author's knowledge, no other work has solely focused on reconstructing historical trade and GDP statistics for as many island territories as possible.

There are at least three important methodological challenges the author has faced. The first one has been knowing what the colonial administration have produced in terms of reports and yearly accounts. If a certain year has not been registered or accounted for by the colonial administration at the time, the data cannot probably be reconstructed. It is important as one has to know what they are looking for so as to not waste time and resources searching for reports that were never produced (or were destroyed throughout history).

The second challenge, after understanding what was produced by whom, was to actually identify where the archival material was physically held. The INSEE Archives (Bibliothèque Desrosières) was extensively visited and data were found with the help of local archivist Thierry Couderc. The challenge lies in assembling data from various international archival funds. Indeed, the author contacted the national archives and/or statistical offices of every former colonial power (UK, USA, The Netherlands, Germany, Portugal, Spain, Danemark, Italy) as well as a lot of local (island) statistical offices. The latter usually have less material as the yearly colonial reports were sent to the central colonial administrations.

The third challenge was the impact of political status on data availability. Indeed, independent island territories have their data published yearly and are systematically included in international databases. They also tend to have colonial documents that are easier to access and sometimes held by the local national archives. On the other side, islands that are still affiliated to a metropolitan power are usually excluded from these databases. Only the most autonomous ones, with some form of local self-government, are included in the aforementioned databases. As one can understand, there is an important self-selection biais if one were to select and study only island territories available in international databases and already published materials. The innovation this data paper brings is the systematic inclusion and study of island territories, regardless of their political status.

The data collection process took a lot of calibration with plenty of trial and error. Since mid-2021, the author systematically looked for some form of ‘annual reports’ or ‘colonial reports’, which feature imports, exports and population data, when international databases or other researchers' published data were lacking or insufficient. This was done to reconstruct data prior to the 1950′s, for both affiliated and independent islands, if they were not already covered by Bulmer-Thomas or Mitchell.

The UNCTAD publishes data going back to 1948 with a rather large and comprehensive scope, as it includes not only independent but also very autonomous territories. The author also consulted every single issue of the UN Statistical Yearbook from the 1st to the 50th issue. Some island territories are covered there and not covered by the UNCTAD. Using the three first issues, the author has found data going back to 1929 and 1931 for some territories. Prior to these years (1929-1931), all data coming from territories not covered by either Bertram [1], Bulmer-Thomas [2] or Mitchell [4] were found, reported and compiled by the author. There have also been verifications (when possible) made by the author regarding locally reported data and data available in international databases or scholarly works. Overall, the degree of confidence is very good as data are very close. Thus, when available, data coming from international databases were used as the series were easily available in a continuous manner since 1948 for UNCTAD data, since 1960 for WDI GDP data and since 1970 for UN Statistics Department.

It is important to note that the exploration was much larger than what appears in this dataset. Some islands were integrated to the national polity for a long time and thus were not thought of as different from any other part of the national territory. A good illustration of this remark would be the Greek, Croatian and British island territories. Indeed, for exemple Isle of Man, Isle of Wight, Jersey and Guernsey are all British islands that have been integrated to Britain for multiple centuries, thus they have historically not counted trade coming from and going to Britain as international trade, rather it was counted as internal trade, as would be trade between two regions of mainland Britain. The same can be said for Croatian, Greek, French, Australian, Indonesian, …, Islands.

After having contacted all the aforementioned statistical offices and archival funds, the author was fundamentally limited by what was digitized and available online. Thus, the decision was made to go to the British archives (National Archives at Kew and British Library, London). This research trip was useful insofar it provided an overview of what was available in both institutions. The author has consulted and reported all data found in Annual and Colonial Reports for various British crown colonies and protectorates. Unfortunately, the islands that were under the Western Pacific High Commission (Solomon Islands, Gilbert and Ellice Islands, New Hebrides, Tonga, Pitcairn) were not available as they have been progressively sent to the University of Auckland special Collections.

## Limitations

There are several limitations with this work. The first one is data availability. Indeed, other data sources could have been included in this dataset, especially in the ‘*NLucic_SIHTD_Details_of_Foreign_Trade_per_Island_with_sources-06032024*′. Some sources could not be explored as they have to be physically consulted. For as much as the author is aware, the Western Pacific Archive held by the University of Auckland, New Zealand, holds the archival data for island territories that were under British colonial control or protectorate. Also, data for Sardinia and Sicily, since the late 19th, can be found in the physical Istat Biblioteca in Rome.^4^ As only a very small part of the total material held in the fund has been digitized, one has to physically explore these archives. The author did not have time nor funding for this endeavor.

One other major limitation is the construction of the inflation index as it is at the core of the transformed data. That said, researchers are welcome to use more disaggregated parts of the dataset.

## Ethics Statement

The author, Nicolas Lucic, has read and followed the ethical requirements for publication in Data in Brief. This work does not include any human subjects, animal experiments nor is any data collected from social media platforms.

## CRediT Author Statement

The author is the sole contributor to this work. Every part of the CRediT statement, from Conceptualization to project administration was done by Nicolas Lucic.

## Data Availability

SIHTD - Small Island Historical Trade Statistics (Original data) (Recherche.Data.gouv.fr -> Paris-Saclay)

## Footnotes

1 Available here: databank.worldbank.org/source/world-development-indicators

2 Available here : https://data.un.org/Data.aspx?d=SNAAMA&f=grID%3A101%3BcurrID%3AUSD%3BpcFlag%3A1%3BcrID%3A92

3 Available here : https://www.cambridge.org/fr/universitypress/subjects/history/economic-history/economic-history-caribbean-napoleonic-wars?format=PB&isbn=9780521145602#resources

## Footnotes

4 Address : Via Cesare Balbo 16, Rome, Italy, website: https://www.istat.it/it/informazioni-e-servizi/per-gli-utenti/biblioteca
